# Use of navigation channels by Lake Sturgeon: Does channelization increase vulnerability of fish to ship strikes?

**DOI:** 10.1371/journal.pone.0179791

**Published:** 2017-07-05

**Authors:** Darryl W. Hondorp, David H. Bennion, Edward F. Roseman, Christopher M. Holbrook, James C. Boase, Justin A. Chiotti, Michael V. Thomas, Todd C. Wills, Richard G. Drouin, Steven T. Kessel, Charles C. Krueger

**Affiliations:** 1 Great Lakes Science Center, U.S. Geological Survey, Ann Arbor, MI, United States of America; 2 Great Lakes Science Center-Hammond Bay Biological Station, U.S. Geological Survey, Millersburg, MI, United States of America; 3 Alpena Fish and Wildlife Conservation Office-Waterford Substation, U.S. Fish and Wildlife Service, Waterford, MI, United States of America; 4 Lake St. Clair Fisheries Research Station, Michigan Department of Natural Resources, Harrison Township, MI, United States of America; 5 Lake Erie Management Unit, Ontario Ministry of Natural Resources and Forestry, London, Ontario, Canada; 6 Department of Fisheries and Wildlife, Michigan State University, East Lansing, MI, United States of America; 7 Center for Systems Integration and Sustainability, Michigan State University, East Lansing, MI, United States of America; Pacific Northwest National Laboratory, UNITED STATES

## Abstract

Channelization for navigation and flood control has altered the hydrology and bathymetry of many large rivers with unknown consequences for fish species that undergo riverine migrations. In this study, we investigated whether altered flow distributions and bathymetry associated with channelization attracted migrating Lake Sturgeon (*Acipenser fulvescens*) into commercial navigation channels, potentially increasing their exposure to ship strikes. To address this question, we quantified and compared Lake Sturgeon selection for navigation channels vs. alternative pathways in two multi-channel rivers differentially affected by channelization, but free of barriers to sturgeon movement. Acoustic telemetry was used to quantify Lake Sturgeon movements. Under the assumption that Lake Sturgeon navigate by following primary flow paths, acoustic-tagged Lake Sturgeon in the more-channelized lower Detroit River were expected to choose navigation channels over alternative pathways and to exhibit greater selection for navigation channels than conspecifics in the less-channelized lower St. Clair River. Consistent with these predictions, acoustic-tagged Lake Sturgeon in the more-channelized lower Detroit River selected the higher-flow and deeper navigation channels over alternative migration pathways, whereas in the less-channelized lower St. Clair River, individuals primarily used pathways alternative to navigation channels. Lake Sturgeon selection for navigation channels as migratory pathways also was significantly higher in the more-channelized lower Detroit River than in the less-channelized lower St. Clair River. We speculated that use of navigation channels over alternative pathways would increase the spatial overlap of commercial vessels and migrating Lake Sturgeon, potentially enhancing their vulnerability to ship strikes. Results of our study thus demonstrated an association between channelization and the path use of migrating Lake Sturgeon that could prove important for predicting sturgeon-vessel interactions in navigable rivers as well as for understanding how fish interact with their habitat in landscapes altered by human activity.

## Introduction

Channelization is a common river engineering practice and disturbance of river ecosystems that has been associated with declines in native fish biodiversity and fishery productivity [1–4]. Channelization is the straightening and deepening of rivers, which increases channel flow capacity and current velocity and improves conditions for vessel navigation and for flood control [1, 3, 5]. Most studies addressing channelization effects on fish have focused on channelization as a disturbance that negatively affects local habitat conditions for fish. Indeed, numerous studies have documented that fish abundance and species diversity is lower in channelized reaches than in adjacent reaches unaltered by dredging, bank hardening, and/or riverbed armoring [6–8]. Comparatively few studies have examined channelization as a factor that influences how fish interact with their habitat despite growing evidence that animals may exhibit non-optimal behaviors in human-dominated landscapes [9].

This study examined the potential of channelization to influence fish migratory behavior with implications for fish conservation and future navigational engineering in large rivers. Fish species that undergo riverine migrations as adults or juveniles often navigate using hydraulic cues such as current direction, current velocity, and discharge [10–14]. Channelization therefore has the potential to influence path choice of migrating fish if individuals prefer the higher flows or swifter currents of channelized river sections over the hydraulic characteristics of alternative pathways. Fish attracted to channelized pathways that coincide with shipping routes may be injured or killed as a result of collisions with commercial vessels [15, 16].

Field studies designed to evaluate the effects of channelization and river engineering practices on fish movements and vulnerability to vessel strikes can be confounded by the presence of locks or dams that prevent fish from accessing pathways alternative to main navigation channels. When alternative corridors are present, studies comparing fish use of navigational channels to unaltered river sections can reveal associations that might inform strategies to mitigate potential fish-vessel interactions [17]. However, systems suitable for such comparative work are rare due to the high incidence of locks and dams in the mainstem sections of most of the world’s large rivers [18].

The goal of the present study was to compare Lake Sturgeon (*Acipenser fulvescens*) use of alternative movement pathways in two rivers differentially affected by navigational engineering but free of barriers to fish movement (i.e., no mainstem locks or dams) to determine if channelization has the potential to increase fish vulnerability to ship strikes by attracting migrating individuals into commercial navigation channels. Lake Sturgeon inhabit large freshwater lakes and rivers of central North America and migrate into rivers for the purposes of spawning, feeding, and/or overwintering [19–21]. Lake sturgeon were once abundant throughout their native range, but due to overfishing and habitat fragmentation, the species is currently listed as threatened or endangered in eleven U.S. states and in seven Canadian provinces [19]. Under the assumption that Lake Sturgeon navigate by following primary flow paths, we predicted that 1) migrating individuals would select the higher-flow, faster-moving, and deeper navigation channels over alternative pathways within a defined reach of a more-channelized river and that 2) Lake Sturgeon selection for navigation channels would be greater in the more-channelized river than in the less-channelized river. If true, then Lake Sturgeon in channelized rivers could be at increased risk of ship strikes and require additional protection.

## Materials and methods

### Study site

**Fig 1:**
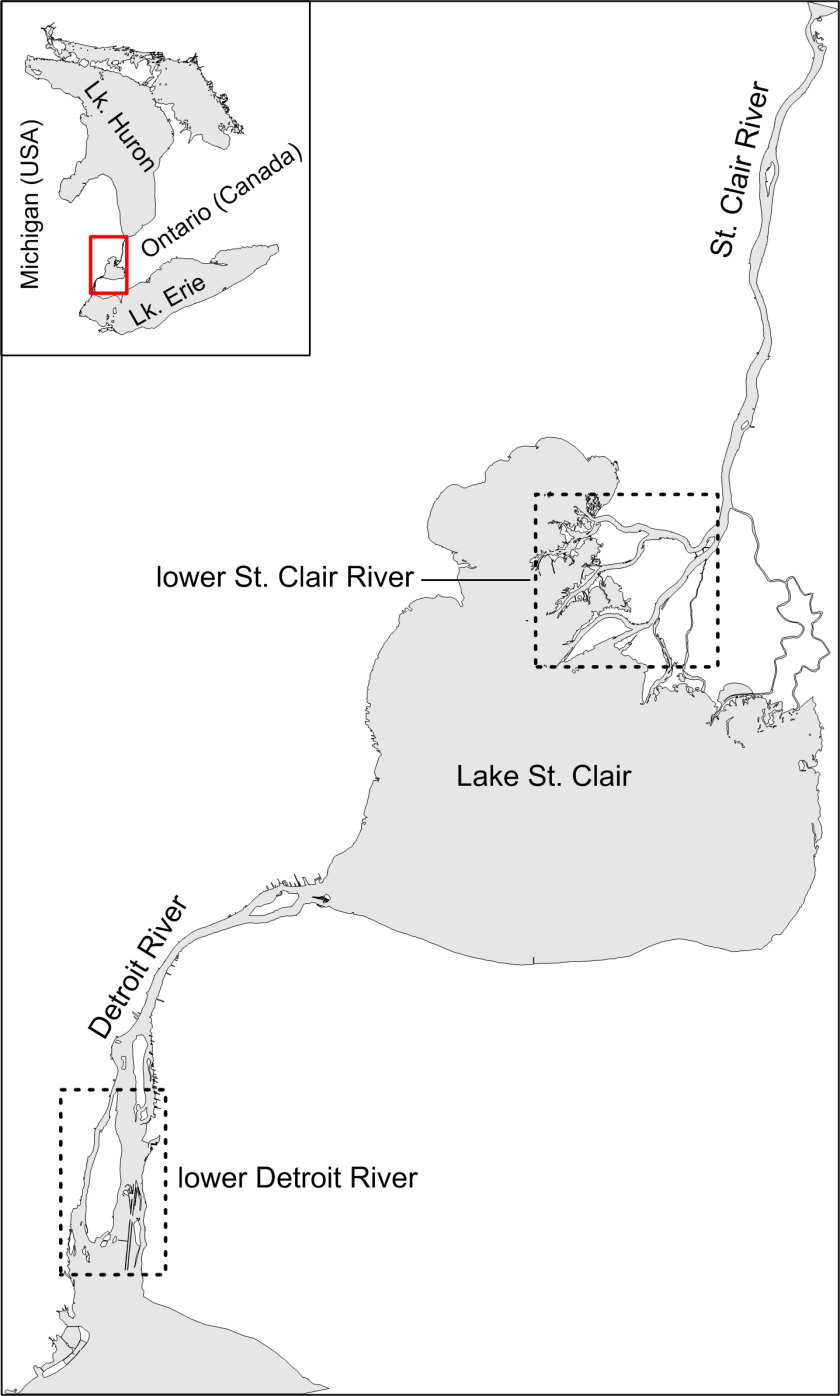
The Detroit-St. Clair River system. Dashed boxes delineate the extent of the lower Detroit and lower St. Clair rivers. The direction of flow is from Lake Huron to Lake Erie (i.e., from north to south).

**Fig 2:**
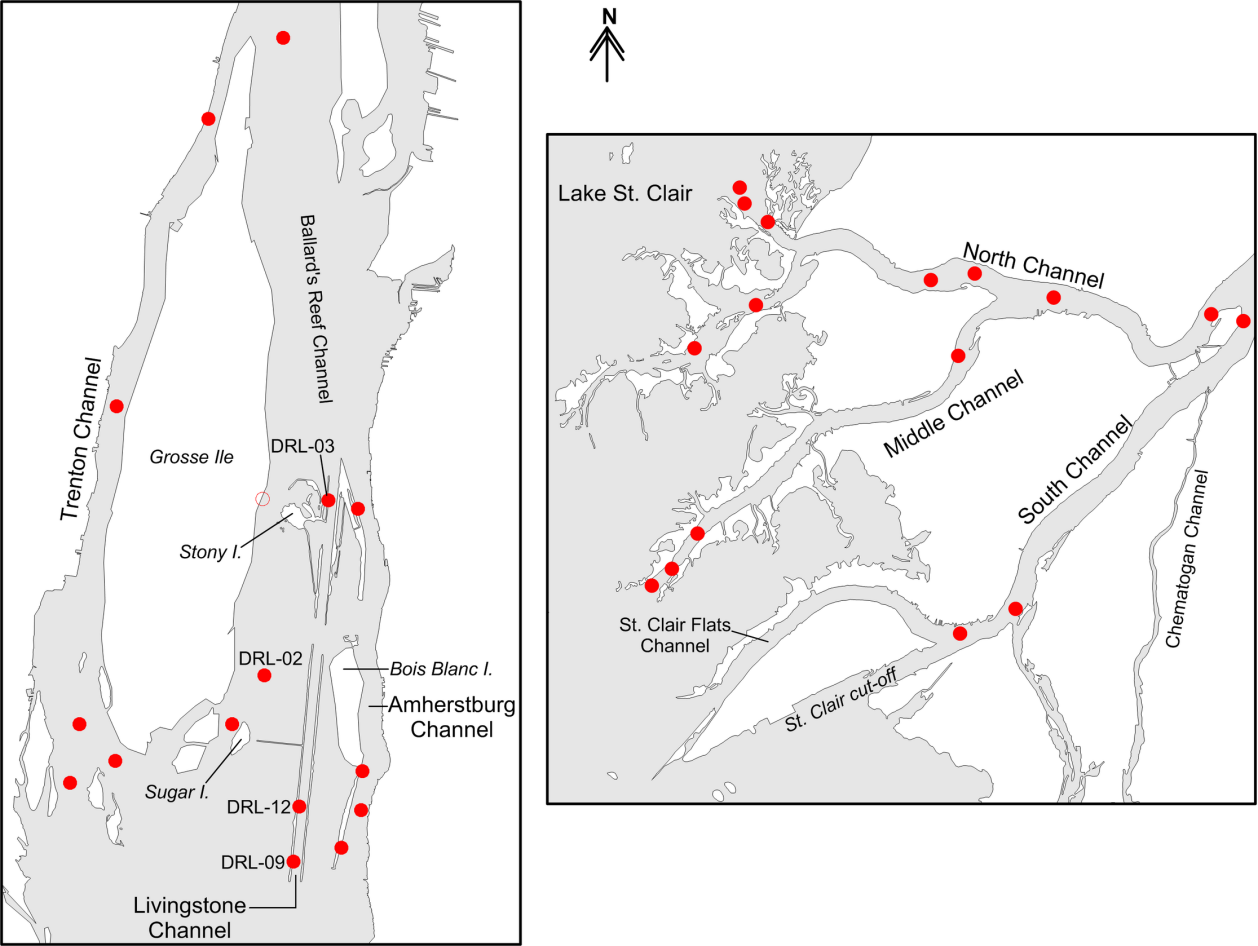
The lower Detroit River (left panel) and lower St. Clair River (right panel). Red circles show locations of acoustic receivers. The unfilled circle shows the location of station DRL-02 in 2012. Beginning in 2013, DRL-02 was moved to its current location (filled red circle with text “DRL-02”).

Effects of channelization on the paths used by migrating Lake Sturgeon were explored in the lower Detroit and St. Clair rivers, which together with Lake St. Clair, comprise the waterway connecting Lake Huron and Lake Erie (Fig 1). The lower Detroit River was defined as the river section between the upstream tip of Grosse Ile (42.20202, −83.14215) and the downstream end of the Amherstburg and Livingstone channels (42.06837, −83.12759), and the lower St. Clair River was defined as the network of channels between the upstream tip of Russell Island (42.61550, −82.52241) and Lake St. Clair (Figs 1 and 2). Pathways for migrating Lake Sturgeon in the lower Detroit River included the Trenton Channel, the channel between Sugar Island (42.09128, −83.14436) and Stony Island (42.12631, −83.13256), the Livingstone Channel, and the Amherstburg Channel. The Sugar I.-to-Stony I. pathway encompassed the waters east of Grosse Ile and west of the Livingstone navigation channel (Fig 2). In the lower St. Clair River, the primary pathways for sturgeon movement were the North, Middle, and South channels (Fig 2). Length of the individual channels ranged from 5.6 km (Sugar I.-to-Stony I. pathway) to 13.9 km (Trenton Channel). Average channel width ranged from 172 m (Livingstone Channel) to 909 m (Sugar I.-to-Stony I. pathway), although the hydrologically-effective portions of the channels often were much narrower due to large expanses of shallow water (depth < 3.7 m) present at the margins of all the channels except for the Livingstone Channel. Except for a small section of the upper Trenton Channel, all ship and freighter traffic is restricted to the Livingstone and Amherstburg navigation channels of the lower Detroit River and to the South Channel of the lower St. Clair River (Fig 2). The St. Clair cut-off at the downstream end of the South Channel enables ships to bypass the shallow, winding St. Clair Flats Channel (Fig 2).

The Detroit-St. Clair river system is part of a busy international waterway. Commercial (merchant) ships and freighters make approximately 5,000 trips though each lower river each year (Canadian Vessel Traffic Management Information System-INNAV database; http://www.innav.gc.ca/home.aspx; queried 18 August 2016). The primary commercial vessels using the navigation channels are cargo-carrying ships and freighters (e.g., bulk carriers, self-unloaders, tug-and-barge units, and tankers). The sizes of domestic cargo-carrying ships in the Great Lakes are large relative to the size of the navigation channels. For instance, the largest domestic bulk carriers are 305 m in length and have beams that are nearly 20% the width of the Livingstone Channel. Propellers of bulk carriers and self-unloaders vessels can range up to 5.3 m in diameter and may extend to within a meter of the bottom in channelized river sections.

The Detroit-St. Clair river system supports a large remnant population of Lake Sturgeon. Size of the St. Clair River-Lake St. Clair population segment was estimated at approximately 45,000 adults, although this number may have included some individuals from the Detroit River and Lake Huron [22]. Lake Sturgeon migrate into the lower Detroit and St. Clair rivers to spawn, feed, and/or overwinter [23–25].

**Fig 3:**
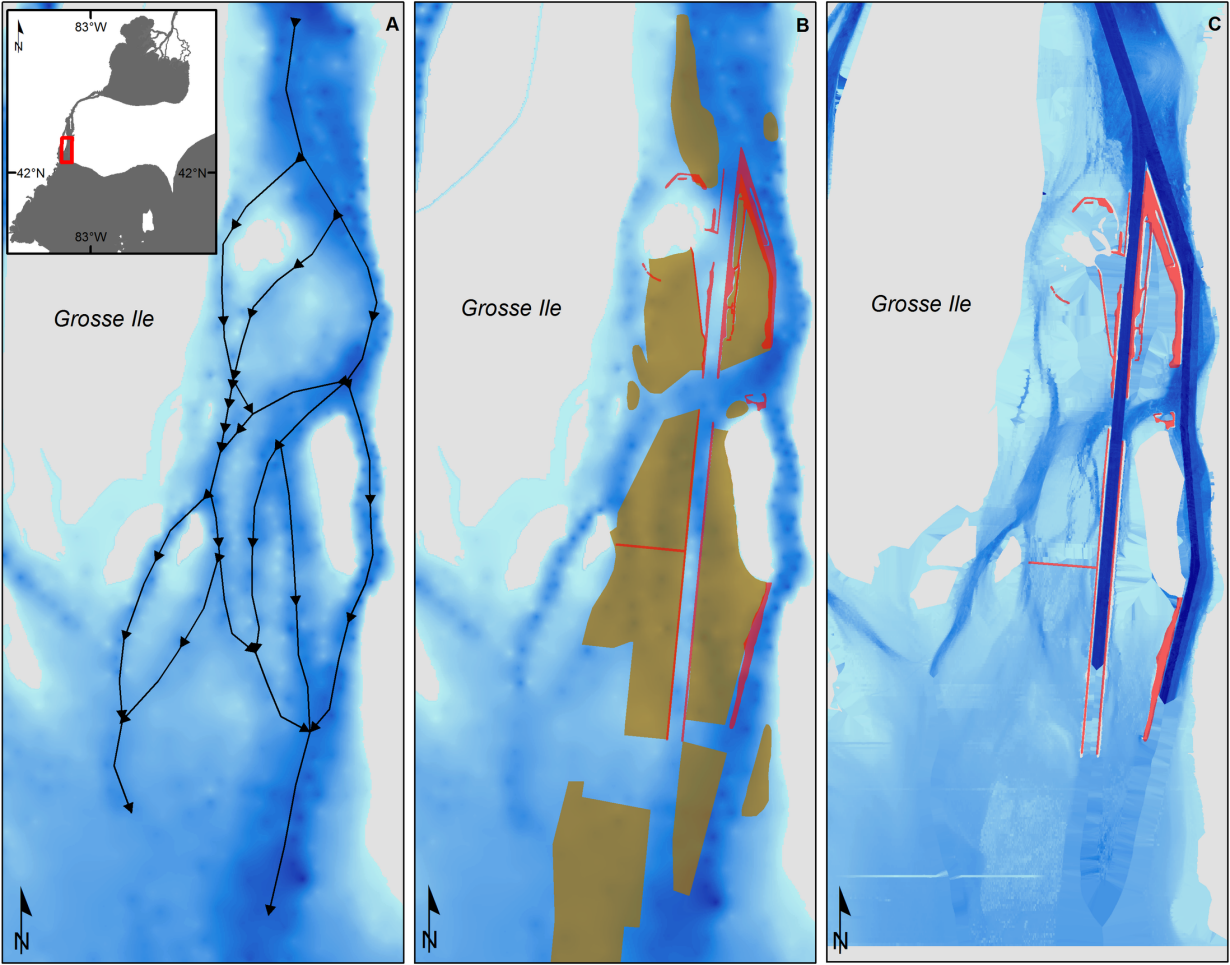
Bathymetry of the lower Detroit River east of Grosse Ile before (A, B) and after (C) channelization (circa 1900 vs. 2012). Black arrows indicate potential pathways for fish movement. Brown-shaded areas show dredge spoil disposal locations. Above-water compensating works are shown in red. Bathymetry data for the lower Detroit River in 2012 was provided by the U.S. Army Corps of Engineers-Detroit District.

**Fig 4:**
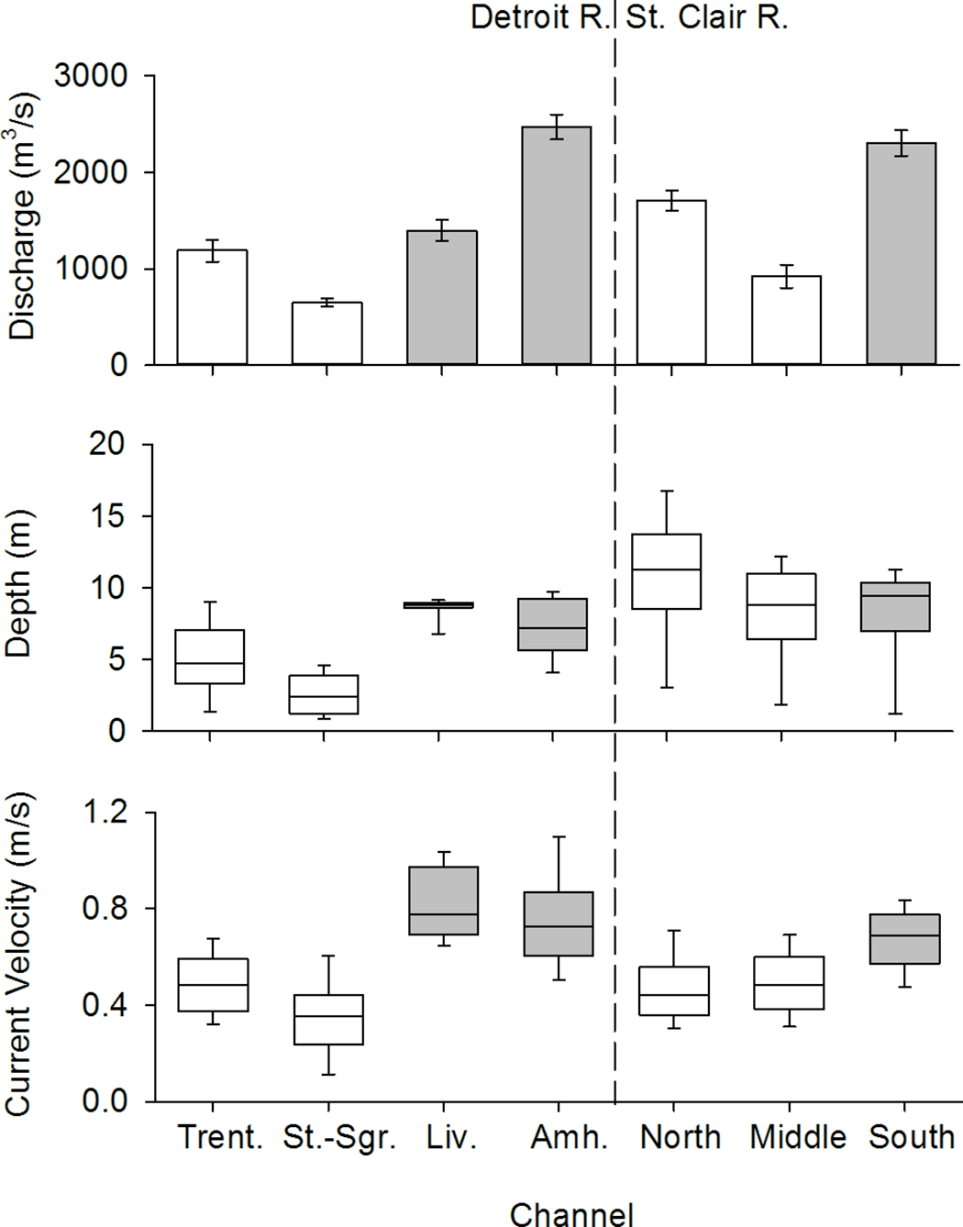
Current hydraulic and bathymetric characteristics of navigation channels (shaded) and other main channels in the lower Detroit and St. Clair rivers. Trent. = Trenton Channel, St.-Sgr. = Sugar I.-to-Stony I. channel, Liv. = Livingstone Channel, Amh. = Amherstburg Channel, *Upper panel*: mean annual discharge (± 95% confidence interval). Data from Table 3 of Holtschlag and Koschik [27]. *Middle panel*: Variation (median and quartiles) in channel depth. Error bars represent the 10th and 90th percentiles. Data provided by the U.S. Army Corps of Engineers-Detroit District. *Lower panel*: Variation (median and quartiles) in channel depth-averaged current velocity. Data provided by the NOAA-Great Lakes Environmental Research Laboratory. Error bars represent the 10th and 90th percentiles.

The lower Detroit River has been more altered by navigational engineering and channelization than the lower St. Clair River. Prior to 1868, the lower Detroit River to the east of Gross Ile consisted of a network of relatively shallow channels that were difficult for large, deep-draft vessels to navigate (Fig 3A). During 1900–1968, construction of the new Livingstone navigation channel and deepening of the existing Amherstburg channel improved the navigability of the lower Detroit River, but generated 2.50×10^8^ m^3^ of dredge spoil that was subsequently dumped into adjacent channels [26] (Fig 3B and 3C). The combination of shipping channel construction and dredge spoil disposal also forced much the river’s flow to the east of Bois Blanc Island (42.09666, −83.12102) and through the Amherstburg Channel [26]. The only major navigation engineering project in the lower St. Clair River was the 1960–1962 construction of the St. Clair cut-off. Presently, navigation channels in the lower Detroit River tend to have greater flow, depth, and current velocity than other channels, whereas these variables vary less among the channels of the lower St. Clair River (Fig 4).

### Acoustic telemetry

Acoustic telemetry was used to describe the path choice of migrating Lake Sturgeon in the lower Detroit and St. Clair rivers. In acoustic telemetry, fish are captured, surgically-implanted with an acoustic transmitter (or ‘tag’), and subsequently released back into the environment. Archival acoustic hydrophones or ‘receivers’ then are used to detect and record acoustic signals from tagged individuals. Acoustic telemetry was an ideal tool for documenting route choice of Lake Sturgeon in the lower Detroit and St. Clair rivers because it allowed for concurrent monitoring of tagged individuals at multiple sites.

Between 2011 and 2015, a total of 282 adult-sized Lake Sturgeon (total length 117.4–180.3 cm) were captured in the Detroit and St. Clair rivers, and in Lake Huron, and surgically implanted with an acoustic transmitter (Vemco 69 kHz V16-6L; power output = 152 dB re 1μPa at 1m). The acoustic transmitters had a projected battery life of 10 years, were 16 mm in diameter, 95 mm long, and weighed 34 g in air. All fish were captured between late April and early July. Following recovery from surgery, acoustic-tagged Lake Sturgeon were released back into the environment near (±0.5 km) the point of capture. Lake Sturgeon capture and tagging procedures were described in detail by Hondorp, Holbrook, & Krueger [28]. Collection of Lake Sturgeon in the Detroit and St. Clair rivers was authorized by the Michigan Department of Natural Resources (Threatened and Endangered Species Permit 1932) and the Ontario Ministry of Natural Resources and Forestry (Lake Huron collector’s permit UGLMU2015-03 and Endangered Species Permit AY-B-008-12).

Sampling and handling of Lake Sturgeon in this study conducted in accordance with guidelines for the care and use of fishes in research developed by the American Fisheries Society, the American Society of Fishery Research Biologists, and the American Society of Ichthyologists and Herpetologists [29]. Lake sturgeon were not anesthetized prior to surgery because a proven and widely-accepted method for field anesthetization of large fish was not available at the time of this study (but see [30] for a new electronarcosis method). Instead, individuals implanted with acoustic tags were immobilized ventral-side up in a mesh sling with head and gills immersed in a 379 liter (100 US gallons) tank continuously supplied with river water. Lake Sturgeon under these conditions exhibited behaviors consistent with tonic immobility, which likely minimized the stress of handling and surgery. The authors certify that intracoelomic implantation of acoustic tags in Lake Sturgeon followed surgical best practices as recommended by Cooke et al. [31] and Wagner et al. [32].

Movements of acoustic-tagged Lake Sturgeon through the channels and distributaries of the lower Detroit and St. Clair rivers were monitored on a network of 31 Vemco 69 kHz VR2W receivers (Detroit R. = 15, St. Clair R. = 16; Fig 1). Acoustic receivers were moored to the river bottom (with hydrophone directed upwards) as described by Hondorp, Holbrook, & Krueger [28]. Receivers recorded the date, time, and unique ID code for each acoustic tag detection. Placement of receivers at the ends of channels enabled us to determine the timing of channel entrance and exit, as well as the direction of sturgeon movement (upstream vs. downstream). Probability of acoustic-tagged Lake Sturgeon passing individual receivers undetected was estimated to be less than 9% [28]. Acoustic detection data were downloaded from receivers biannually and uploaded to a central database maintained by the Great Lakes Acoustic Telemetry Observation System (GLATOS; www.data.glos.us/glatos/). The data set available for analysis included all available detections through September 2016.

### Acoustic detection data processing and analysis

Sequences of locations and corresponding detection dates/times (herein referred to as “detection histories”) were provided by GLATOS from their central database for each acoustic-tagged Lake Sturgeon. Detection histories were screened for potential false positives according to the procedure described by Hondorp, Holbrook, & Krueger [28]. A total of 23,713 detections (< 0.1% of all detections) were identified as potential false positives and removed from the data set. Repeated detections of an individual on the same receiver that occurred within one hour were collapsed into detection “events.” Event time was the median time of the constituent detections. Detection events then were used to define movements of Lake Sturgeon herein referred to as “passages.” A passage was defined as the movement of an acoustic-tagged Lake Sturgeon through the entire length of an individual channel or distributary that was completed in less than 2 days. Adult-sized Lake Sturgeon (total length ≥ 120 cm) swimming at a sustainable swimming speed of 96.8 cm s−^1^ [33] are capable of traversing the entire length of any channel in the lower Detroit or St. Clair Rivers in less than 4 hours; thus, individuals requiring 2 days or longer to traverse these channels were considered to be engaged in behaviors other than migration (e.g., feeding or spawning). Passages were aggregated by river (Detroit, St. Clair), channel (Amherstburg, Livingstone, Sugar I.-to-Stony I., Trenton, North, Middle, and South channels), channel type (navigation, alternative), and direction (upstream, downstream). Subsequent analyses of Lake Sturgeon channel use and path choice were based on the frequency and distribution of Lake Sturgeon passages.

We were unable to determine directional movements through the Livingstone Channel of the lower Detroit River after October 2013 due to the loss of receivers at stations DRL-03 and DRL-09 in the spring of 2014 (Fig 2). Examination of detection data from 2012 and 2013 showed that 78% of Lake Sturgeon passages (25 of 32) that occurred in the Livingstone Channel required less than two days to complete; thus, in subsequent years (2014–16), we estimated passage frequency in the Livingstone Channel under the assumption that each detection event at the remaining station (DRL-12) corresponded to a single passage event. Data analyses were repeated with and without the estimated Livingstone Channel passage data to ensure that results did not depend on data from missing receivers.

### Data analysis

A generalized linear mixed model (GLMM) was used to evaluate our predictions concerning the effects of channelization on the use of alternative pathways by migrating Lake Sturgeon. The binary response variable indicated whether an individual Lake Sturgeon passage was observed in a navigation channel (= 1) or an alternative channel (= 0). A logit link function was selected because the response variable was binary. The model predicted the relative probability that an individual Lake Sturgeon passage occurred in a navigation channel vs. an alternative pathway (i.e., another channel not used for commercial navigation). Fixed effects in the model included river (Detroit vs. St. Clair), direction of sturgeon movement (upstream vs. downstream), and their interaction. River was used as proxy variable for the extent of channelization, which was greater in the lower Detroit River than in the lower St. Clair River. Movement direction was included in the model because the number of available pathways and their configuration differed depending on whether sturgeon were moving upstream or downstream. A sturgeon-specific random intercept was added to the model to compensate for potential correlation among passages made by the same individual. The model of interest included main effects for river, direction, and their interaction. Mixed model analyses were performed using PROC GLIMMIX in SAS 9.4 [34]. Explanatory power of the GLMM model was assessed from conditional *R*^2^, which describes the proportion of total variance explained by the combination of fixed and random effects [35]. Conditional *R*^2^ [*R*^2^_GLMM(*m*)_] was estimated using function ‘sem.model.fits’ in package ‘piecewiseSEM’ in R [36]. The significance level (α) was set to 0.05 for all analyses.

To determine if Lake Sturgeon in the more-channelized lower Detroit River selected navigation channels over alternative pathways (prediction 1), the probability of navigation channel use (from the GLMM model) was compared to expected use under the null hypothesis that Lake Sturgeon use of different channel types would be proportional to their combined flow (i.e., discharge). If the hydrology and bathymetry of channelized river sections attracts migrating fish, we expected that Lake Sturgeon use of navigation channels in the lower Detroit River would be significantly higher than what would be predicted from flow alone. In contrast, we expected that channel selection by Lake Sturgeon in the less-channelized St. Clair River would more closely approximate null model predictions based on differences in channel discharge. Lake Sturgeon path use was considered significantly different from the null model if the 95% confidence interval for the relative probability of navigation channel use did not encompass the null model prediction. Null models based on differences in depth or current velocity also could have been constructed, but would not yield dramatically different predictions due to the correlation among discharge, depth, and current velocity (see Fig 4).

Hypothesis tests concerning the fixed effects (river, direction, river × direction) were determined from a type-III analysis of the *F*-statistic. If Lake Sturgeon use of navigation channels increased with the extent of channelization (prediction 2), we expected that the relative probability of navigation channel use by Lake Sturgeon would vary significantly between rivers and would be greater in the more-channelized lower Detroit River than in the less-channelized St. Clair River in at least one movement direction.

## Results

### Summary of passage events

**Fig 5:**
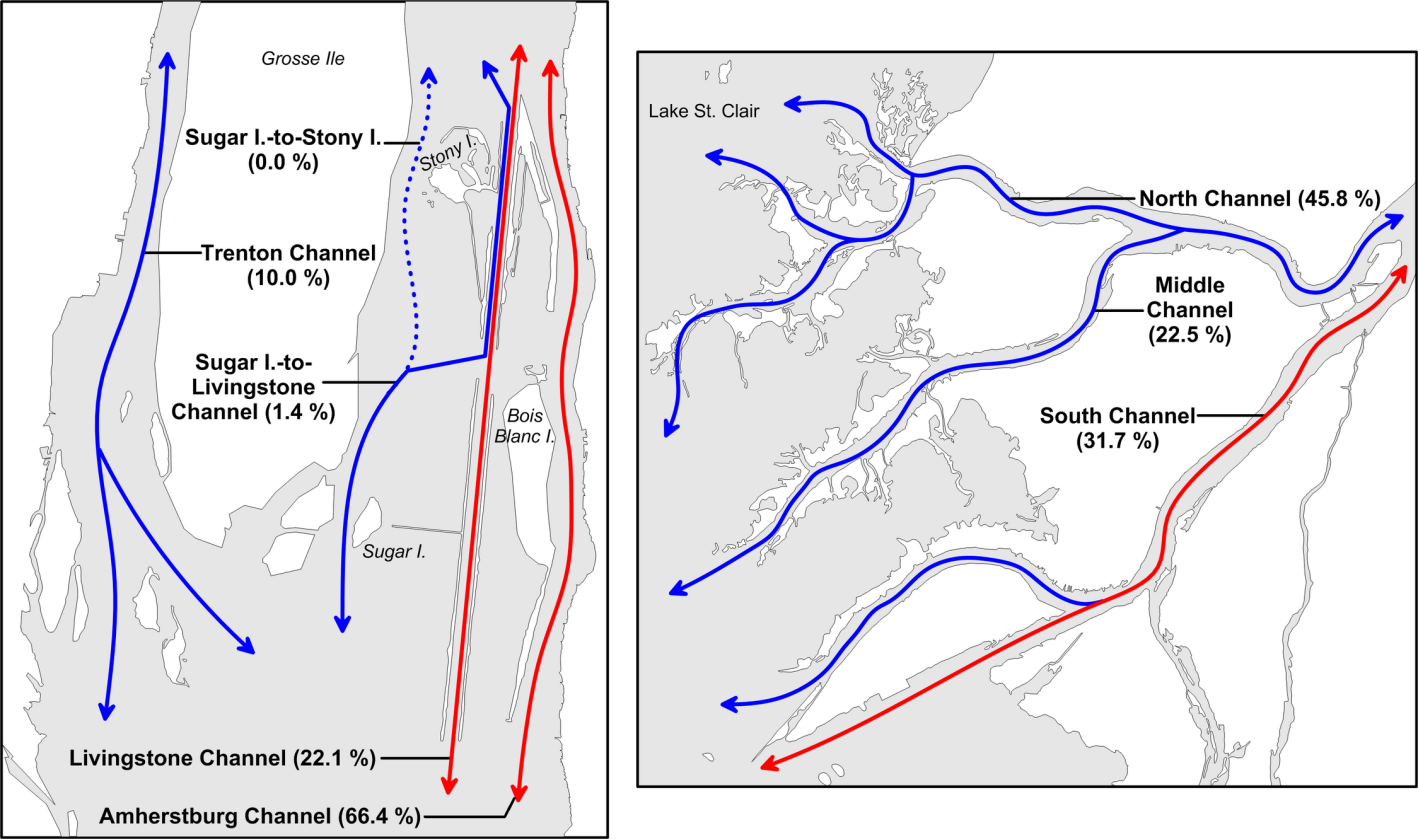
Path use (% of observed passages) by acoustic-tagged Lake Sturgeon in the lower Detroit (left panel) and St. Clair (right panel) rivers. Total number of passages was 280 in the lower Detroit River and 404 in the lower St. Clair River. Movement pathways through navigation channels are shown in red. Lake Sturgeon moving upstream at Sugar Island in the lower Detroit River turned eastward and entered the Livingstone navigation channel (solid path) rather than continuing along the expected path (dashed extension) towards Stony Island.

Analysis of detection data yielded 684 passage events (Detroit = 280; St. Clair = 404) from 186 acoustic-tagged Lake Sturgeon. By direction, 380 passages were classified as downstream, and 269 passages were classified as upstream. After the loss of receivers at stations DRL-03 and DRL-09, we estimated that 35 Lake Sturgeon passages occurred in the Livingstone Channel. This estimate seemed reasonable given that mean annual passage frequency in the Livingstone Channel was similar before (~12.5/year) and after (~11.7/year) loss of the two receivers. Data collected during 2012 suggested that acoustic-tagged Lake Sturgeon did not use the shallow Sugar I.-to-Stony I. channel in the lower Detroit River; thus, the receiver at station DRL-02 was moved to a deeper location about 3.5 km downstream (see Fig 2). However, four passages were observed in which Lake Sturgeon moved between Sugar Island and the upstream portion of the Livingstone channel via the gap in the compensating works (dotted path in Fig 5).

### Effects of river and movement direction on Lake Sturgeon path use

**Fig 6:**
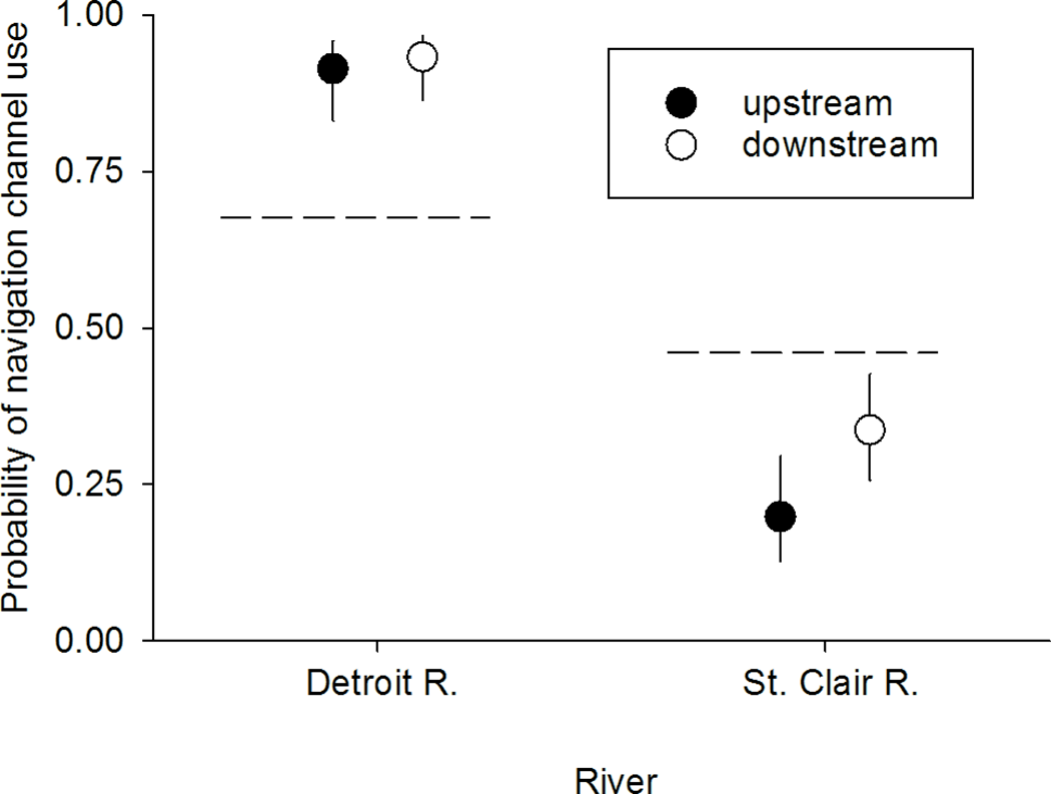
Probability (mean±95% confidence interval) of navigation channel use by acoustic-tagged Lake Sturgeon by river (Detroit vs. St. Clair) and direction of movement (upstream vs. downstream). Expected probabilities of navigation channel use under the null model are shown as dashed horizontal lines.

Consistent with the first prediction, acoustic-tagged Lake Sturgeon selected the deeper and higher-flow navigation channels to alternative pathways in the more-channelized Detroit River, but not in the less-channelized lower St. Clair River. Over 85% of Lake Sturgeon passages in the lower Detroit River occurred in navigation channels compared with 32% in the lower St. Clair River (Fig 5). The probability of a Lake Sturgeon passing through a navigation channel vs. an alternative pathway in the lower Detroit River also was significantly greater than the null model prediction based on channel flow (*P* < 0.05; Fig 6), whereas in the lower St. Clair River, the probability of navigation channel use by acoustic-tagged Lake Sturgeon was less than the null model prediction (*P* < 0.05; Fig 6).

**Fig 7:**
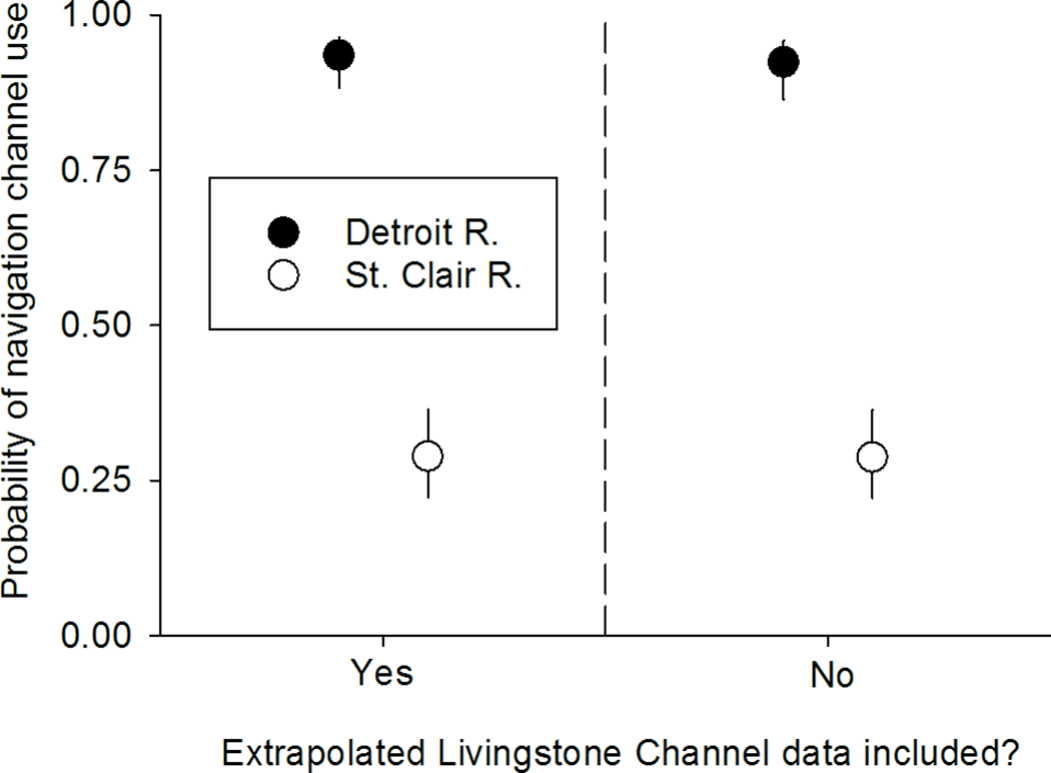
Probability (mean±95% confidence interval) of navigation channel use by acoustic-tagged Lake Sturgeon in the lower Detroit and St. Clair rivers when analyses included and excluded the extrapolated Livingstone Channel passage data. Upstream and downstream passages were pooled because direction of sturgeon movement for the extrapolated sturgeon passage data was unknown. Expected probabilities of navigation channel use under the null model are shown as dashed horizontal lines.

Consistent with the second prediction, Lake Sturgeon selection for navigation channels as migration routes was greater in the more-channelized lower Detroit River than in the less-channelized lower St. Clair River. Probability of acoustic-tagged Lake Sturgeon using navigation channels vs. alternative routes differed significantly between rivers (GLMM; river effect; *df* = 1,460, *F* = 53.45, *P* < 0.01) and was three to four times higher in the lower Detroit River than in lower St. Clair River (Fig 6). The effect of river on the probability of navigation channel use did not depend on whether Lake Sturgeon were moving upstream or downstream (GLMM; river × direction effect; *df* = 1,460, *F* = 0.81, *P* = 0.37; Fig 6). Probability of navigation channel use by acoustic-tagged Lake Sturgeon was only 11% higher for downstream-moving individuals than for upstream-moving individuals, but this difference was significant (GLMM; direction effect; *df* = 1,460, *F* = 6.67, *P* = 0.01). The preceding inferences were considered relatively robust to the effects of un-modeled influences on Lake Sturgeon movement given that the GLMM model explained nearly 60% of the total variance in Lake Sturgeon movement data (*R*^2^_GLMM(*m*)_ = 0.57). Trends in Lake Sturgeon path use also were unaffected by the inclusion of passage frequencies estimated for the Livingstone Channel during 2014–2016 (Table 1, Fig 7).

**Table 1:** Path use (% of observed passages) by acoustic-tagged Lake Sturgeon in the lower Detroit River including and excluding the extrapolated Livingstone channel passage data.

	Extrapolated Data Included?	
Channel or Pathway	Yes	No
Trenton	10.0	11.4
Sugar I.-to-Stony I.	1.4	1.6
Livingstone*	22.1	11.0
Amherstburg*	66.4	75.9

*Navigation channels

## Discussion

Use of navigation channels by Lake Sturgeon in the lower Detroit and St. Clair rivers was consistent with the hypothesis that migrating fish prefer the greater depths, higher flows, and/or swifter currents of channelized river sections over the hydraulics and bathymetry of alternative pathways. In support of this hypothesis, Lake Sturgeon in the more-channelized lower Detroit River showed strong positive selection for navigation channels over alternative pathways, whereas Lake Sturgeon in the less-channelized lower St. Clair River selected alternative pathways to the main navigation channel. Selection for navigation channels by migrating Lake Sturgeon also was three to four times higher in the more-channelized lower Detroit River than in the less-channelized lower St. Clair River.

**Fig 8:**
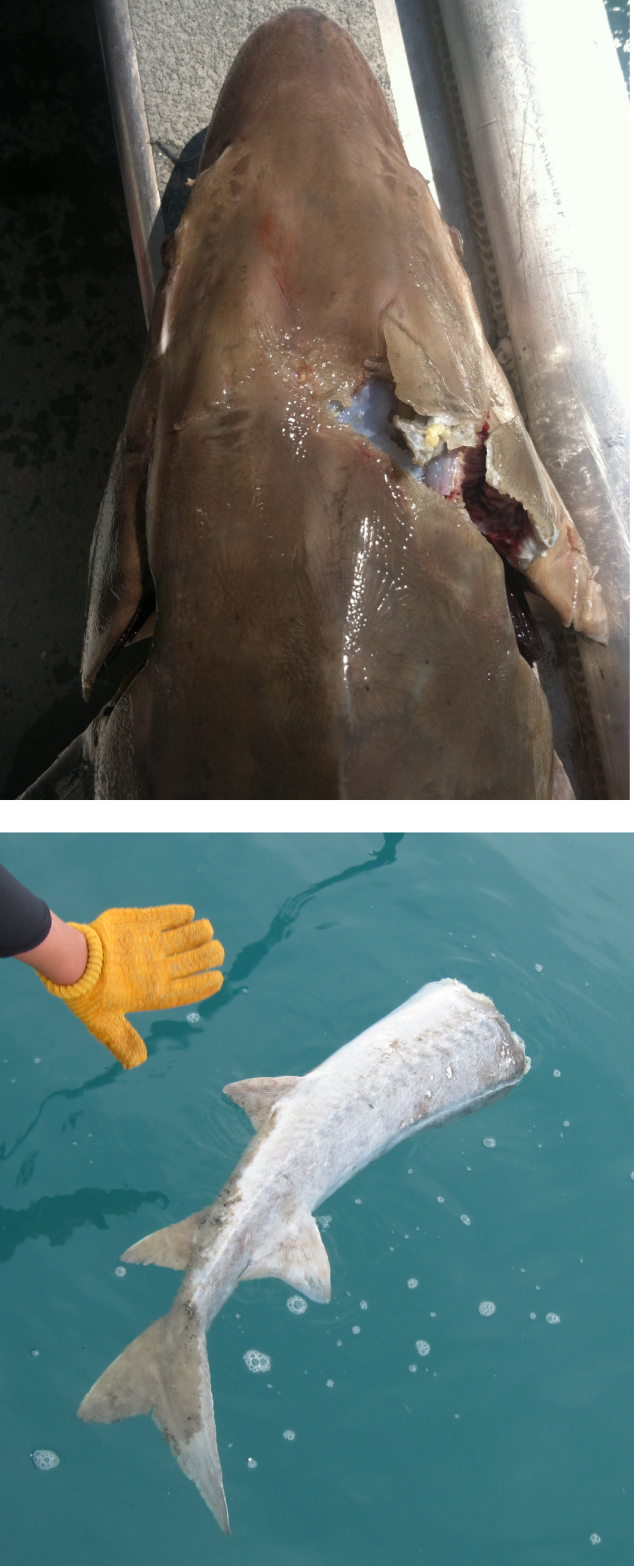
Photographs of injured and dead Lake Sturgeon with wounds consistent with vessel propeller strikes. *Upper panel*: Lake Sturgeon with fractured skull and operculum photographed in the Livingston Channel of the lower Detroit River on 13 May 2014 (photo credit: P.A. Thompson). *Lower panel*: Decapitated Lake Sturgeon carcass photographed in the South Channel of the lower St. Clair River on 10 June 2010 (photo credit: D.W. Hondorp).

Lake Sturgeon selection of navigation channels over alternative pathways in the lower Detroit River supported the contention that channelization of rivers increases vulnerability of sturgeon to ship strikes by increasing spatial overlap of sturgeon with commercial vessels. Lake sturgeon within navigation channels likely follow the same paths as commercial vessels given previous research that showed that adults in the St. Clair River rarely use habitats with depths less than the controlling (minimum) navigation depth of 8.2 m [23, 37]. The large size (length, beam, draft) of commercial vessels relative to the scale of the channels (see methods) also suggests that relatively little space exists at preferred depths for lake sturgeon to avoid commercial vessels in shipping lanes. Our study did not quantify ship-strike mortality for acoustic-tagged Lake Sturgeon, but we have observed injured and dead Lake Sturgeon bearing wounds consistent with propeller hits in the navigation channels of the lower Detroit and St. Clair rivers (Fig 8). Ship-strikes involving sturgeon also have been reported in the Mississippi, Illinois, Delaware, James and Yangtze rivers [15, 16, 38–41]. Miranda and Killgore (40) estimated that the combined mortality from fishing and ship-strikes for shovelnose sturgeon (*Scaphirhynchus platorhynchus*) in the Mississippi river was unsustainable, which demonstrated that negative interactions with commercial vessels have the potential to threaten sturgeon recovery. Control of adult mortality is a cornerstone of Lake Sturgeon recovery plans in the Great Lakes Basin, so fishery managers unaware of the potential for ship-strike mortality may assume that all anthropogenic sources of mortality for adult Lake Sturgeon have been adequately controlled through restrictions on commercial and recreational fishing.

The hypothesized link between channelization and sturgeon vulnerability to ship strikes also is consistent with the results of theoretical and empirical work showing that animal movements in human-altered landscapes can be non-optimal and potentially maladaptive [9]. Non-optimal movements occur when an animal’s use of formerly-adaptive behaviors in human-modified landscapes expose it to hazards that threaten its survival [9, 42]. For example, in our study, Lake Sturgeon that we presume were navigating according to historically reliable hydraulic cues selectively used commercial navigation channels as movement corridors in a channelized river ecosystem (lower Detroit River), potentially increasing their risk for negative interactions with commercial vessels. By influencing fish path use, channelization also could attract migrating fish into the turbines of hydropower facilities or cause spawning-ready individuals to bypass historic spawning sites, thereby delaying breeding activity or inducing use of unsuitable spawning habitat. Special regulations (e.g., alteration of shipping routes or restrictions on commercial vessel operating speeds in navigation channels) may be required to protect migratory fish exhibiting non-optimal behaviors in large rivers.

Our conclusions regarding the effects of channelization on path choice of migrating Lake Sturgeon are subject to the caveat that the pre-channelization movements of Lake Sturgeon in the lower Detroit River were unknown. Thus, Lake Sturgeon may have always preferred to migrate and disperse through the part of the Detroit River now occupied by the navigation channels. However, the presence of historical Lake Sturgeon spawning sites to the west of the area currently occupied by the navigation channels [43] suggests that the movements of spawning-ready individuals in the lower Detroit River historically were more distributed among the available channels than observed in this study. Another potential criticism of our study is that it was observational and unreplicated. We acknowledge that additional studies similar to the one performed here would help to validate our results, but reach-scale manipulation of flow distributions, channel morphology, and commercial vessel traffic are not possible in most systems. Given this constraint, our experimental design, which involved examining Lake Sturgeon movements within an existing gradient of environmental stress (channelization), was a sound and reasonable approach. The gradient-response study design used here also has been successfully implemented to examine commercial vessel traffic as a disturbance that affects the distribution of riverine fish [17].

Our conclusions about the effects of channelization on fish migratory behavior also depend on the assumption that Lake Sturgeon use hydraulic cues during river migration and navigate by following primary flow paths. We contend this assumption is justified given the results of recent field studies showing that fish ranging from salmonids (*Oncorhynchus* spp.) to eels (*Anguilla* spp.) use information on river discharge as well as current velocity and acceleration to make decisions about path choice [13, 44–46]. While comparable studies with sturgeon have not been conducted, the reported relationship between river discharge and the timing of Lake Sturgeon spawning runs in the Black River system (Michigan, USA) [47] suggests that Lake Sturgeon are capable of detecting variation in hydraulic variables. Many fish species also use olfaction to home to natal habitats in rivers [48], but the use of chemosensory cues for route selection in the lower Detroit and St. Clair Rivers seems unlikely given that over 95% of the water flowing through Detroit-St. Clair river system emanates from a single source (Lake Huron). The migrations of long-lived fish like Lake Sturgeon migrations also could be guided or aided by social learning and spatial memory, mechanisms known to influence habitat use and feeding behavior of sharks [49, 50]. Given that the only known Lake Sturgeon spawning site in the lower St. Clair River was located in the North Channel, spatial memory or learning from more experienced individuals could potentially explain why Lake Sturgeon use of the navigation channel in the lower St. Clair River was significantly lower than the null prediction based on channel discharge. However, learning and spatial memory also could act to reinforce patterns of movement driven by responses to hydraulic variables.

In conclusion, results from the present study demonstrated an association between channelization and the path use of migrating Lake Sturgeon that could prove to be important for understanding sturgeon-vessel interactions in navigable rivers. The main evidence for this association was that Lake Sturgeon use of navigation channels as pathways for movement was significantly higher in the more-channelized lower Detroit River than in the less-channelized lower St. Clair River. Selection for navigation channels by Lake Sturgeon in the lower Detroit River suggested that migrating fish chose the hydraulics and bathymetry of channelized river sections over the physical characteristics of alternative pathways. We therefore speculated that channelization promotes use of pathways (e.g., commercial navigation channels) that increases fish exposure to threats to which they are not adapted (e.g., ship strikes). Non-optimal movements by fish likely have been overlooked in many systems, but might be common due to the pervasiveness of channelization in large rivers. If so, evaluation of proposed river engineering projects should consider not only potential effects on fish habitat, but also the potential of engineering practices such as channelization to influence how fish interact with their habitat, perhaps with negative consequences.
